# ﻿New species of *Urodeta* Stainton, 1869 (Lepidoptera, Elachistidae, Elachistinae) from Ghana and Democratic Republic of the Congo, with identification keys to the Afrotropical species of the genus

**DOI:** 10.3897/zookeys.1089.79716

**Published:** 2022-03-11

**Authors:** Virginijus Sruoga, Jurate De Prins

**Affiliations:** 1 Life Sciences Centre of Vilnius University, Saulėtekio str. 7, LT-10257 Vilnius, Lithuania Life Sciences Centre of Vilnius University Vilnius Lithuania; 2 Royal Belgian Institute of Natural Sciences, 1000 Brussels, Belgium Royal Belgian Institute of Natural Sciences Brussels Belgium

**Keywords:** Microlepidoptera, mining moths, morphology, Sub-Saharan Africa, taxonomy

## Abstract

Two new species, *Urodetafalcata***sp. nov.** from Ghana and *U.bisigna***sp. nov.** from Democratic Republic of the Congo are described. The habitus and genitalia are diagnosed and illustrated in detail. Identification keys to the Afrotropical species of the genus *Urodeta*, based on male and female genitalia, are provided.

## ﻿Introduction

The genus *Urodeta* was established by Stainton (1869) with *U.cisticolella* Stainton, 1869 as the type species. Originally, Stainton (1869) indicated its closeness to *Elachista* Treitschke, but subsequent classifications have associated it with several different families and subfamilies (De Prins and Sruoga 2012).

Moths of the genus *Urodeta* are very small to small with a wingspan of 4–8 mm. The labial palpus is porrect and shorter than the diameter of the head. The forewing pattern is mostly inconspicuous, being unicolourous or with indistinct markings. The most distinctive feature in the male genitalia is the anteriorly directed spines of the gnathos, and females are easily recognized by the apophyses anteriores, which, when present, extend from the middle of segment 8 and spread apart laterad. A more detailed list of the morphological characters diagnosing this genus have been summarized and verified by Kaila (2004, 2011) and Sruoga and De Prins (2011, 2013). The known larvae are leaf-miners in dicotyledonous plants in the families *Cistaceae* (Stainton 1869; Lhomme 1946–1963; Zerkowitz 1946) and *Combretaceae* (Kaila 2011).

Until 2009, *Urodeta* was thought to be monotypic and its distribution restricted to the Mediterranean region. Taxonomic interest in this genus increased following the description of a considerable number of new species from tropical Africa (Mey 2007; Sruoga and De Prins 2009, 2011; De Prins and Sruoga 2012), Australia (Kaila 2011) and Asia (Sruoga and De Prins 2013; Sruoga and Rocienė 2018; Sruoga et al. 2019). The genus *Urodeta* now comprises 26 accepted and validly named species (Kaila 2019) distributed in Europe, Africa, Asia and Australia, but most of the species are known from tropical Africa (Table 1). Kaila (2011) recognized one additional species, but did not name it.

**Table 1.** *Urodeta* species and their distributions.

*Urodeta* species	Distribution	Notes	References
*hibernella* (Staudinger, 1859)	Mediterranean Region	Male and female	Staudinger (1859); Bengtsson (1997); Koster and Sinev (2003)
*falcata* sp. nov.	Ghana	Male only	Present study
*absidata* Sruoga & De Prins, 2011	Cameroon	Male and female	Sruoga and De Prins (2011)
*aculeata* Sruoga & De Prins, 2011	Cameroon	Male only	Sruoga and De Prins (2011)
*crenata* Sruoga & De Prins, 2011	Cameroon	Male only	Sruoga and De Prins (2011)
*cuspidis* Sruoga & De Prins, 2011	Cameroon	Male only	Sruoga and De Prins (2011)
*faro* Sruoga & De Prins, 2011	Cameroon	Female only	Sruoga and De Prins (2011)
*tortuosa* Sruoga & De Prins, 2011	Cameroon	Female only	Sruoga and De Prins (2011)
*acerba* Sruoga & De Prins, 2011	Democratic Republic of Congo	Male and female	Sruoga and De Prins (2011)
*bisigna* sp. nov.	Democratic Republic of Congo	Female only	Present study
*bucera* Sruoga & De Prins, 2011	Democratic Republic of Congo	Male and female	Sruoga and De Prins (2011)
*talea* Sruoga & De Prins, 2011	Democratic Republic of Congo	Male and female	Sruoga and De Prins (2011)
*falciferella* (Sruoga & De Prins, 2009)	Kenya	Female only	Sruoga and De Prins (2009)
*gnoma* (Sruoga & De Prins, 2009)	Kenya	Male only	Sruoga and De Prins (2009)
*spatulata* (Sruoga & De Prins, 2009)	Kenya	Male and female	Sruoga and De Prins (2009)
*tantilla* (Sruoga & De Prins, 2009)	Kenya	Male only	Sruoga and De Prins (2009)
*maculata* (Mey, 2007)	Namibia	Male and female	Mey (2007)
*taeniata* (Mey, 2007)	Namibia	Male only	Mey (2007)
*acinacella* Sruoga & De Prins, 2012	South Africa	Female only	De Prins and Sruoga (2012)
*quadrifida* Sruoga & De Prins, 2012	South Africa	Female only	De Prins and Sruoga (2012)
*trilobata* Sruoga & De Prins, 2012	South Africa	Male and female	De Prins and Sruoga (2012)
*jurateae* Sruoga & Rocienė, 2018	India	Male and female	Sruoga and Rocienė (2018)
*pectena* Sruoga & Rocienė, 2018	India	Female only	Sruoga and Rocienė (2018)
*noreikai* Sruoga & De Prins, 2013	Nepal	Male and female	Sruoga and De Prins (2013)
*longa* Sruoga & Kaila, 2019	Thailand	Female only	Sruoga et al. (2019)
*inusta* Kaila, 2011	Australia	Male and female	Kaila (2011)
*Urodeta* sp.	Australia	Described, but not named; male and female	Kaila (2011)

In this study, we describe two new species in the genus *Urodeta* and provide keys to all the known Afrotropical species.

## ﻿Materials and methods

Adult specimens were examined externally using MBS-10 and Euromex Stereo Blue stereomicroscopes. The forewing length was measured along the costa from wing base to the apex of the terminal fringe scales. For a wingspan, the forewing length was doubled and thorax width added. The width of the head was measured between the inner edges of the antennal bases. Genitalia were prepared following the standard method described by Robinson (1976) and Traugott-Olsen and Nielsen (1977). The genitalia were studied and some morphological structures were photographed in glycerol before permanent slide-mounting in Euparal. The male genital capsule was stained with fuchsin and the abdominal pelt with chlorazol black (Direct Black 38/Azo Black). The genital morphology was examined using a Novex B microscope. Habitus images were taken using a Canon EOS 80D camera fitted with a MP-E 65 mm Canon macro lens, attached to a macro rail (MJKZZ Qool Rail). The photographs of genitalia were made using a Novex B microscope and a E3ISPM12000KPA digital camera. The descriptive terminology of morphological structures follows Kaila (1999, 2011) and Kristensen (2003).

Type specimens are deposited in the Royal Belgian Institute of Natural Sciences, Belgium (**RBINS**).

## ﻿Taxonomy

### ﻿Key to the Afrotropical species of *Urodeta* species based on male genitalia

[males of the following species are unknown and not included in the key: *U.bisigna* sp. nov., *U.falciferella*, *U.quadrifida* and *U.tortuosa*]

**Table:** 

1	Sacculus entirely separated from remaining valva as an elongate lobe	**2**
–	Sacculus not separated from remaining valva	**3**
2	Valva divided into two separate lobes (sacculus and remaining part of valva); sclerotized phallic tube not dilated basally (Sruoga and De Prins 2011, figs 25–28)	***U.acerba***
–	Valva divided into three distinct lobes (sacculus entirely separated and termen of remaining part of valva deeply emarginated so appear divided into long and narrow lobes); sclerotized phallic tube strongly dilated basally (De Prins and Sruoga 2012, figs 22 and 23)	***U.trilobata***
3	Ventral margin of sacculus partly serrated (Sruoga and De Prins 2011, figs 52–55)	***U.crenata***
–	Ventral margin of sacculus not serrated	**4**
4	Spinose knob of gnathos divided into two separated lobes (Sruoga and De Prins 2011, figs 39–41)	***U.bucera***
–	Spinose knob of gnathos not divided	**5**
5	Inner processes of valvae fused apically and embedded with many small cusp-shaped spines (Sruoga and De Prins 2011, figs 15–20)	***U.absidata***
–	Valva without inner process embedded with spines	**6**
6	Phallus with strongly sclerotized band along ventral margin	**7**
–	Phallus without strongly sclerotized band along ventral margin	**11**
7	Valvae are tightly fused together dorso-proximally (Sruoga and De Prins 2011, figs 74–76)	***U.talea***
–	Valvae not fused together dorso-proximally	**8**
8	Indentation of distal margin of juxta wider than width of juxta lobe (Sruoga and De Prins 2011, figs 35 and 36)	***U.aculeata***
–	Indentation of distal margin of juxta is not wider than juxta lobe or juxta not indented	**9**
9	Vesica with a cluster of small internal spines and two large, claw-shaped cornuti (this paper, Figs 4, 6–7, 9, and 10)	***U.falcata***
–	Vesica with a cluster of small internal spines and more than two large cornuti	**10**
10	Vesica with a cluster of small internal spines and four large cornuti (Mey 2007, figs 33 and 34)	***U.maculata***
11	Sclerotized phallic tube about 7 times longer than wide; vesica without cornuti (Mey 2007, figs 35 and 36)	***U.taeniata***
–	Sclerotized phallic tube 3.5–5 times longer than wide; vesica with few large cornuti and many tiny internal spines	**12**
12	Vesica with one large cornuti and with group of minute spines (Sruoga and De Prins 2011, figs 58–63)	***U.cuspidis***
–	Vesica with more than one large cornuti and can be with group of minute spines	**13**
13	Sacculus meeting cucullus at sharp angle (about 50–80°); apex of phallus pointed (Sruoga and De Prins 2009, figs 37, 39, and 40)	***U.gnoma***
–	Sacculus meeting cucullus at blunt angle (about 110–145°); apex of phallus with broad, strongly sclerotized process (Sruoga and De Prins 2009, figs 44–47)	***U.spatulata***

### ﻿Key to the Afrotropical species of *Urodeta* species based on female genitalia

[females of the following species are unknown and not included in the key: *U.aculeata*, *U.crenata*, *U.cuspidis*, *U.falcata* sp. nov., *U.faro*, *U.gnoma*, *U.taeniata*, *U.tantilla*]

**Table:** 

1	Corpus bursae with signum	**2**
–	Corpus bursae without signum	**9**
2	Corpus bursae with two signa (this paper, Fig. 14)	***U.bisigna***
–	Corpus bursae with one signa	**3**
3	Both pairs of apophysis (anterioris and posterioris) present	**4**
–	Apophysis anterioris absent	**7**
4	Ductus bursae not coiled	**5**
–	Ductus bursae coiled (Sruoga and De Prins 2009, figs 41–43)	***U.falciferella***
5	Apophysis posterioris long, more than 9 times longer than wide	**6**
–	Apophysis posterioris very short, about 4.5 times longer than wide (Sruoga and De Prins 2011, figs 42–49)	***U.bucera***
6	Ductus bursae with longitudinal folds; signum sickle-shaped (De Prins and Sruoga 2012, figs 6–10)	***U.acinacella***
–	Ductus bursae without longitudinal folds; signum formed by two weakly connected plates, each with a large spine and few smaller ones (De Prins and Sruoga 2012, figs 14–16)	***U.quadrifida***
7	Signum formed by oval sclerotized plate with one large and several small spines (De Prins and Sruoga 2012, figs 24–28)	***U.trilobata***
–	Signum formed by weakly sclerotized plate with long teeth in row	**8**
8	Ductus bursae coiled; corpus bursae with minute internal spines, signum formed from 6–7 stout teeth (Sruoga and De Prins 2011, figs 77–82)	***U.talea***
–	Ductus bursae not coiled; corpus bursae without minute internal spines, signum formed from 4 stout teeth (Mey 2007, figs 30 and 31)	***U.maculata***
9	Corpus bursae divided by narrow prolonged constriction into two parts (Sruoga and De Prins 2011, figs 29–32)	***U.acerba***
–	Corpus bursae not divided	**10**
10	Corpus bursae narrow and long, about 4 times longer than wide (Sruoga and De Prins 2011, figs 21 and 22)	***U.absidata***
–	Corpus bursae rounded	**11**
11	Antrum with strongly sclerotized longitudinal folds (Sruoga and De Prins 2009, figs 48 and 49)	***U.spatulata***
–	Antrum without strongly sclerotized longitudinal folds	**12**
12	Colliculum about 3 times longer than wide; antrum long and weakly sclerotized (Sruoga and De Prins 2011, figs 66–71)	***U.faro***
–	Colliculum as long as wide; antrum short and strongly sclerotized (Sruoga and De Prins 2011, figs 85–88)	***U.tortuosa***

#### ﻿ Urodeta falcata sp. nov.

Taxon classificationAnimaliaLepidopteraMomphidae

460F2506-5ADD-57C0-93EC-2453FDFE8637

http://zoobank.org/50E30AD5-4F6B-47E5-B9F9-3662FD9350CC

Figs 1 , 2 , 5–14

##### Material examined.

***Holotype*.** Ghana • ♂; Ashanti Bobiri, 4 km NE Kubease, 6°41'N, 1°20'W; 230 m alt.; 25 May 2011; J. & W. De Prins leg., gen. prep. VS510.

##### Diagnosis.

*Urodetafalcata* is a small, dark-coloured species with indistinct wing markings. In wing pattern and male genitalia, the new species is most similar to *U.aculeata* Sruoga & De Prins, 2011, known from Cameroon, *U.tantilla* Sruoga & De Prins, 2011, known from Kenya and *U.maculata* (Mey, 2007), known from Namibia. However, *U.falcata* can be distinguished most easily by the presence of two claw-shaped cornuti, pointed apex of phallus and long ventral shield of juxta.

**Figures 1–4.**
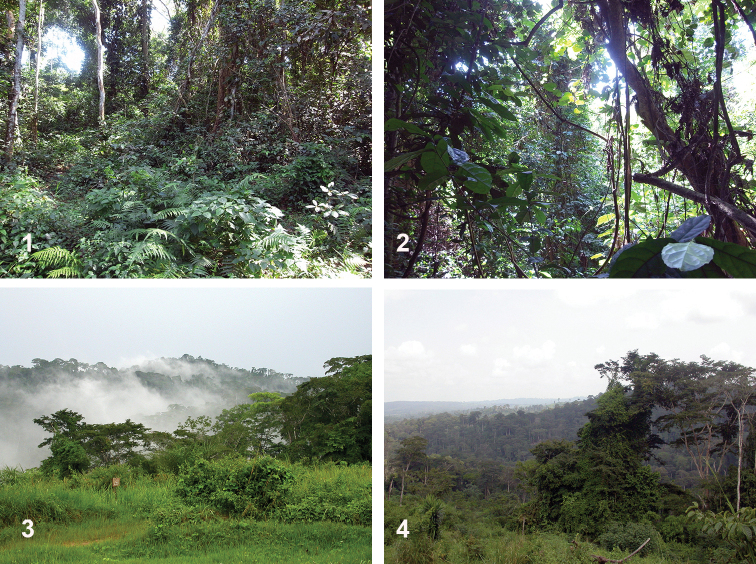
Collecting localities in Sub-Saharan Africa **1, 2** Bobiri Forest, Ashanti, Ghana **3, 4** Mayumbe Forest, Bas-Congo, Democratic Republic of the Congo.

##### Description.

**Figures 5–14.**
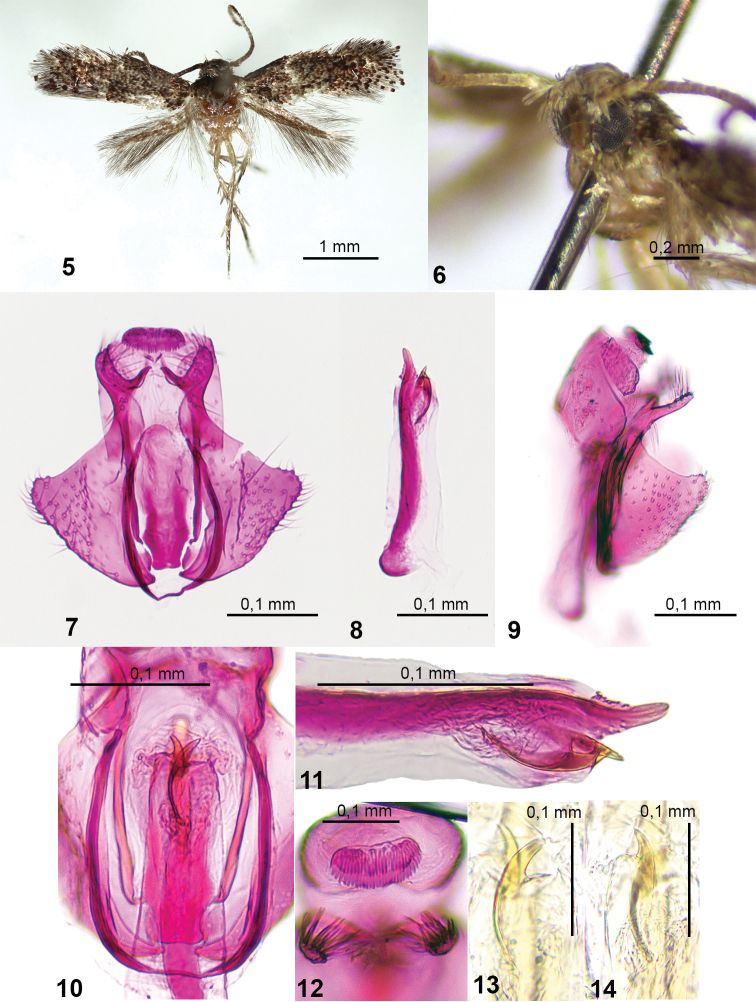
*Urodetafalcata* sp. nov., male, holotype **5** habitus **6** head, fronto-lateral view **7** general view of male genitalia (phallus removed) **8** sclerotized phallic tube **9** male genitalia, lateral view **10** central part of genitalia **11** distal part of phallus **12** gnathos and apices of cucullus, distal view **13** ventral cornutus **14** dorsal cornutus (**5, 6, 8–10** in glycerol before permanent mounting in Euparal).

**Male** (Figs 5, 6). Forewing length 2.2 mm; wingspan 5.0 mm (*N* = 1). ***Head***: frons, vertex and neck tuft pale grey, weakly mottled with dark brown tipped scales; labial palpus vestigial, visible only as very short greyish extension; scape greyish white below, brownish grey above, pecten pale grey; flagellum pale brown, weakly annulated with darker rings basally and slightly serrated apically. ***Thorax*** and tegula strongly mottled with scales basally pale grey and distally brownish grey. Forewing: strongly mottled with scales basally pale grey and distally brownish grey; wing darker beyond middle; fringe brownish grey. Hindwing and its fringe brownish grey.

**Female.** Unknown.

**Male genitalia** (Figs 7–14). Uncus short. Spinose knob of gnathos long oval, twice as long as wide, oriented posteriorly (Fig. 12). Valva short and broad; costa concave; ventral margin of sacculus convex, distally meeting emargination of termen at a blunt angle; cucullus short and narrow, tapered apically, inner surface covered with long setae; transtilla short, strongly sclerotized. Ventral shied of juxta about 3 times as long as wide, strongly sclerotized. Vinculum U-shaped, proximal margin weakly concave. Sclerotized phallic tube short, as long as valva, with strongly sclerotized, wide band along ventral margin; distally tapered towards pointed apex; vesica with 2 large curved cornuti and numerous tiny, elongate spines.

##### Biology.

Unknown.

##### Flight period.

Based on the specimen available, adults fly in May.

##### Distribution.

So far, this species is known only from southern Ghana (Figs 1, 2).

##### Etymology.

The species name is derived from the Latin *falcata* (sickle-shaped) in reference to the shape of cornuti in male genitalia.

##### Remarks.

The head of the holotype is somewhat abraded, therefore the description is approximate.

#### ﻿ Urodeta bisigna sp. nov.

Taxon classificationAnimaliaLepidopteraMomphidae

E490C4F3-64DC-5515-AE42-5097DBE0764C

http://zoobank.org/718EA81F-1BC6-45BA-83DB-0B6F4453571A

Figs 3 , 4 , 15–18

##### Material examined.

***Holotype*.** Congo Dem. Rep. • ♀; Bas-Congo, Nat. Res. Luki-Mayumbe, 05°27'S, 13°05'E; 320 m alt.; 29 Mar. 2006; J. De Prins leg., gen. prep. VS511.

##### Diagnosis.

*Urodetabisigna* is a small, lightly-coloured species, with indistinct wing markings. In female genitalia, the new species is comparable to Afrotropical species with vestigial apophyses and a comb-shaped signum consisting of few stout spines, i.e., *U.maculata* (Mey, 2007) known from Namibia, *U.bucera* Sruoga & De Prins, 2011 and *U.talea* Sruoga & De Prins, 2011, known from Democratic Republic of the Congo. However, *U.bisigna* is distinguished most easily by its additional irregularly shaped signum.

##### Description.

**Figures 15–18.**
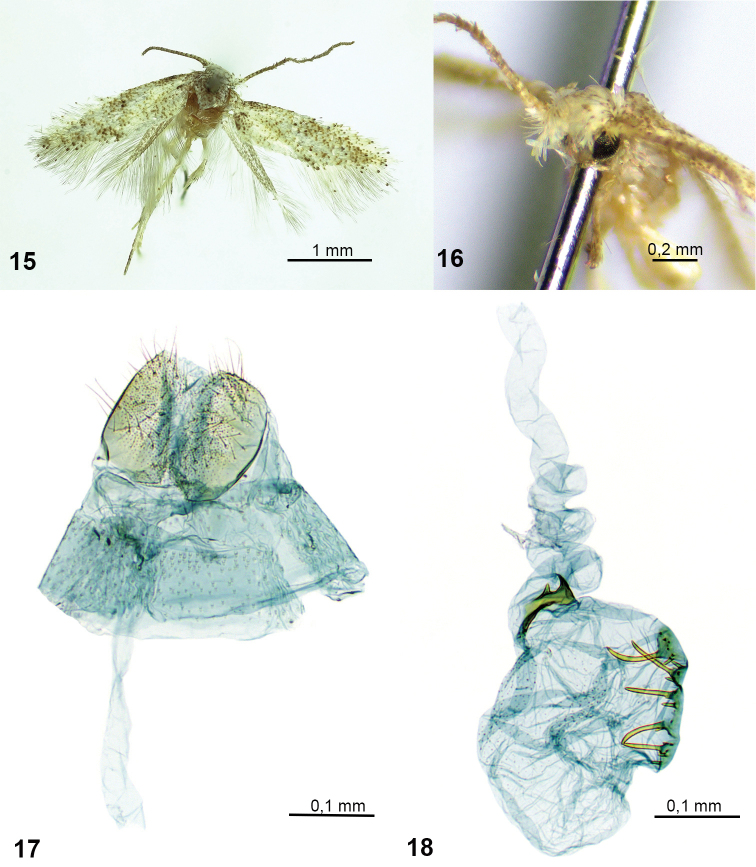
*Urodetabisigna* sp. nov., female, holotype **15** habitus **16** head, fronto-lateral view **17** caudal part of female genitalia **18** ductus and corpus bursae.

**Female** (Figs 15, 16). Forewing length 2.2 mm; wingspan 5.0 mm (*N* = 1). ***Head***: frons, vertex and neck tuft creamy white, neck tuft weakly mottled with brown tipped scales; labial palpus vestigial, visible only as very short greyish extension; scape creamy white, mottled with brown tipped scales above, pecten creamy white; flagellum greyish brown, annulated with paler rings basally and slightly serrated apically. ***Thorax*** and tegula creamy white, mottled by brown tipped scales. Forewing: creamy white powdered with brownish creamy tipped scales. Denser grey brown scales forming two irregular patches: one in basal part of wing; other extending obliquely at 2/5 of costa towards tornus of wing. Blackish brown scales forming two small irregular spots: one at 2/5 of costa and other opposite at dorsum; fringe greyish white. Hindwing and its fringe pale brownish grey.

**Male.** Unknown.

**Female genitalia** (Figs 17, 18). Papilla analis very short, ventral surface setose. Apophysis posterioris vestigial, visible only as tiny extension basolaterally, apophysis anterioris absent. Ostium bursae situated in membrane between sterna 7 and 8. Antrum and colliculum not distinct. Ductus bursae very long, spirally coiled in proximal 1/2. Corpus bursae with minute internal spines and two signa, one comb shaped, consisting of 5 stout teeth, slightly varying in size and few smaller spines; another signum irregularly shaped, with one short spine.

##### Biology.

Unknown.

##### Flight period.

Based on the specimen available, adults fly in March.

##### Distribution.

So far, this species is known only from western Democratic Republic of the Congo (Figs 3, 4).

##### Etymology.

The species name is derived from the Latin prefix *bi* (two), and signum in reference to presence of two signa in female genitalia.

##### Remarks.

The forewing in the holotype is somewhat abraded, therefore the description is approximate.

## ﻿Discussion

In these times of biodiversity loss (De Prins 2022) in Central Africa and elsewhere we recognize the importance of adding two new species for science. The description of two new species brings the total number of known species of Afrotropical *Urodeta* to 20. They comprise nearly 77% of the world fauna of the genus. The largest species richness of *Urodeta* in tropical Africa is reported from Cameroon (6 spp), Democratic Republic of the Congo (4 spp), and Kenya (4 spp). With the description of *Urodetafalcata* sp. nov., the genus *Urodeta* and the subfamily Elachistinae are recorded from Ghana for the first time.

The recent discoveries of *Urodeta* species from Africa, Asia and Australia (Mey 2007; Sruoga and De Prins 2009, 2011, 2013; Kaila 2011; De Prins and Sruoga 2012; Sruoga and Rocienė 2018; Sruoga et al. 2019) show that species richness and geographical distributions are much greater than were previously assumed. The main reason for our limited understanding of this group of moths in the Afrotropical region is a lack of adequate field work. All Afrotropical species of *Urodeta* are known only from their type localities. Although a trend towards endemism of micromoths is evident (De Prins and De Prins 2011–2021), distributions of smaller, more obscure moths might change with targeted collecting efforts outside of the type localities (De Prins et al. 2009).

## Supplementary Material

XML Treatment for Urodeta falcata

XML Treatment for Urodeta bisigna
